# No topoisomerase I alteration in a neuroblastoma model with *in vivo* acquired resistance to irinotecan

**DOI:** 10.1038/sj.bjc.6602079

**Published:** 2004-08-03

**Authors:** L Calvet, A Santos, A Valent, M-J Terrier-Lacombe, P Opolon, J-L Merlin, G Aubert, J Morizet, J H M Schellens, J Bénard, G Vassal

**Affiliations:** 1 Pharmacology and New Treatments in Cancer (UPRES EA 3535), Institut Gustave-Roussy, 39 rue Camille Desmoulins, 94805 Villejuif, France; 2 Oncology Genetics UMR 8125, Institut Gustave-Roussy, Villejuif, France; 3 Department of Pathology, Institut Gustave-Roussy, Villejuif, France; 4 Vectorology and Gene Transfer, UMR 8121, Institut Gustave-Roussy, Villejuif, France; 5 Laboratory of Oncology Research, Centre Alexis Vautrin, Vandoeuvre-Les-Nancy Cedex, France; 6 Department of Medical Oncology, The Netherlands Cancer Institute, Amsterdam, The Netherlands; 7 Laboratory of Molecular Interactions and Cancer, UMR8126, Institut Gustave-Roussy, Villejuif, France

**Keywords:** neuroblastoma, CPT-11, resistance, xenograft

## Abstract

CPT-11 (irinotecan) is a DNA-topoisomerase I inhibitor with preclinical activity against neuroblastoma (NB) xenografts. The aim was to establish *in vivo* an NB xenograft resistant to CPT-11 in order to study the resistance mechanisms acquired in a therapeutic setting. IGR-NB8 is an immature NB xenograft with *MYCN* amplification and 1p deletion, which is sensitive to CPT-11. Athymic mice bearing advanced-stage subcutaneous tumours were treated with CPT-11 (27 mg kg^−1^ day^−1^ × 5) every 21 days (1 cycle) for a maximum of four cycles. After tumour regrowth, a new *in vivo* passage was performed and the CPT-11 treatment was repeated. After the third passage, a resistant xenograft was obtained (IGRNB8-R). The tumour growth delay (TGD) was reduced from 115 at passage 1 to 40 at passage 4 and no complete or partial regression was observed. After further exposure to the drug, up to 28 passages, the resistant xenograft was definitively established with a TGD from 17 at passage 28. Resistant tumours reverted to sensitive tumours after 15 passages without treatment. IGR-NB8-R remained sensitive to cyclophosphamide and cisplatin and cross-resistance was observed with the topoisomerase I inhibitor topotecan. No quantitative or qualitative topoisomerase I modifications were observed. The level of expression of multidrug resistance 1 (MDR1), MDR-associated protein 1 (MRP1) and, breast cancer resistance protein, three members of the ATP-binding cassette transporter family was not modified over passages. Our results suggest a novel resistance mechanism, probably not involving the mechanisms usually observed *in vitro*.

Irinotecan (CPT-11), a semisynthetic water-soluble analogue of camptothecin, belongs to a new family of anticancer drugs, the DNA-topoisomerase I inhibitors. Topoisomerase I is an essential nuclear enzyme that relaxes DNA torsional tension during fundamental processes such as replication, transcription, recombination and repair, and represent a target for many anticancer drugs. Irinotecan settles on the DNA-topoisomerase I complex (cleavable complex), stabilises it and inhibits the religation of DNA. Cytotoxicity arises during the replication process when the advancing replication fork and cleavable complex collide, leading to irreversible DNA damage and to the initiation of a series of events that result in cell death (Chen and Liu, 1994; Pommier, 1996).

Neuroblastoma (NB) is one of the most common solid tumours in young children and is responsible for approximately 7.5% of all cancer among children younger than 15 years of age (Ries *et al*, 1999). It is a neural crest-derived embryonal cancer of the sympathetic nervous system. Biological parameters such as *MYCN* gene amplification (Brodeur *et al*, 1984), loss of heterozygosity of chromosome 1p (Fong *et al*, 1989), (Hayashi *et al*, 1989) diploidy (Look *et al*, 1991) and *MDR1* gene overexpression (Bourhis *et al*, 1989) have been identified as strong predictors of a poor outcome.

CPT-11 has demonstrated antitumour activity against a wide spectrum of both adult and paediatric xenografts in preclinical studies (Komuro *et al*, 1994; Vassal *et al*, 1996; Thompson *et al*, 1997a). We have previously shown that i.v. treatment with CPT-11 resulted in extensive tumour regression and growth delay in three different NB xenograft models (Vassal *et al*, 1996). Similarly, Thompson *et al* (1997b) showed that oral CPT-11 was active against a panel of six NB xenografts. Similar effects have been reported for the topoisomerase I inhibitor topotecan, which induced tumour regression and a significant tumour growth delay (TGD) in animals bearing NB xenografts (Vassal *et al*, 1997). CPT-11 is currently evaluated in children with NB using several doses and schedules, such as 600 mg m^−2^ every 3 weeks, 50 mg m^−2^ day^−1^ × 5 every 3 week or 20 mg m^−2^ day^−1^ × 5 weekly for 2 weeks in a row every 3 weeks (Furman *et al*, 1999; Blaney *et al*, 2001; Vassal *et al*, 2003a).

The emergence of drug resistance in cancer is a major hurdle to successful chemotherapy. Drug resistance has been described in a number of cell lines selected for their resistance to topoisomerase I inhibitors. These studies have shown that quantitative and qualitative changes in topoisomerase I can induce a resistance to topoisomerase I poisons (Li *et al*, 1996; Rubin *et al*, 1996). To date, resistance to CPT-11 has been studied mainly *in vitro*, a situation in which most of the variables are controlled. As this controlled situation cannot be equated with that likely to occur in a therapeutic setting, we established *in vivo* an NB xenograft model resistant to CPT-11, in order to study the mechanisms involved in acquired resistance in this context. It is believed that concrete results thus evidenced can be better translated into clinical applications.

## MATERIAL AND METHODS

### Drugs

CPT-11 was provided by Aventis Pharma SA (Vitry-sur-Seine, France). Cyclophosphamide was purchased from Asta-Medica (Mérignac, France), cisplatin from Bellon and etoposide from Novartis (Rueil-Malmaison, France). Drugs were dissolved in a 0.9% sodium chloride solution immediately before injection on each day of treatment.

### Animals

Female specific pathogen-free Swiss athymic mice (6–8 weeks old) were bred in the Animal Experimentation Unit of the Institut Gustave-Roussy. Animals were housed in sterile isolators and fed with irradiated nutriments (UAR, Villemoisson/Orge, France) and filtered water. Experiments were carried out under the conditions established by the European Community directive no. 86/609/.CEE and in accordance with the UKCCCR guidelines (Workman *et al*, 1998).

### NB xenograft

IGR-NB8 xenograft model was derived from a newly diagnosed stage 3 abdominal NB in a 5-year-old boy, by direct subcutaneous transplantation of small tumour fragments into previously irradiated athymic mice (Vassal *et al*, 1996). The primary tumour of this patient was refractory to conventional chemotherapy that included platinum compounds, cyclophosphamide, doxorubicin, etoposide and vincristine. IGR-NB8 exhibited the classic microscopic appearance of an immature NB. In addition, this model elicited a high tumorigenicity (99%) and a mean tumour doubling time (DT) *in vivo* of 3.3 days. This xenograft displayed the biological features of poor-prognosis NB in children: *MYCN* amplification, near-diploid karyotype and chromosome 1p deletion. In addition, the karyotype showed pericentric inversion of chromosome 2 and additional material on the long arm of chromosome 6. The *MDR1* gene was overexpressed. IGR-NB8 proved to be sensitive *in vivo* to CPT-11, topotecan, cyclophosphamide and cisplatin, but refractory to etoposide (VP16) (Vassal *et al*, 1996; 1997).

### Tumour transplantation

For each experiment, 15–30 mm^3^ tumour fragments were xenotransplanted subcutaneously (unilaterally) into 50 athymic mice. On day 0 of the treatment, mice bearing a 100–300 mm^3^ subcutaneous tumour were randomly assigned to one treated and one control group of five to 10 mice each. Tumour perpendicular diameters were measured twice a week with a caliper, and tumour volume calculated according to the following equation: *V* (mm^3^)=(*d*^2^ (mm^2^) × *D* (mm))/2, where *d* and *D* are the smallest and largest perpendicular tumour diameters, respectively. Each group of mice was treated according to the average weight of the group. Animal body weights were recorded twice weekly and mortality was checked daily. The experiments lasted until tumour volumes reached 1500–2000 mm^3^.

### Treatment

CPT-11 was administered i.v. in a caudal vein at a dose of 27 mg kg^−1^ day^−1^ for 5 consecutive days. This dose was previously shown to induce 100% complete regressions (CR) and to be well tolerated (no treatment-related death and no body weight loss) (Vassal *et al*, 1996). This treatment was repeated every 21 days (one cycle) for a maximum of four consecutive cycles (one passage). During the establishment of *in vivo* resistance, the treatment was stopped either after the fourth cycle or when 50% of the tumours had reached a volume that was five-fold the initial volume. After tumour regrowth following discontinuation of treatment, tumour fragments were xenotransplanted subcutaneously into a new set of 50 athymic mice and the treatment was started according to the same methodology.

Four anticancer compounds were studied to evaluate cross-resistance phenotypes. Topotecan was administered i.p. daily × 5 at a dose of 3.2 mg kg^−1^ day^−1^. Etoposide was administered i.v. daily × 5 at a dose of 20 mg kg^−1^ day^−1^. Cyclophosphamide was administered as a single i.p. injection at a dose of 400 mg kg^−1^. Cisplatin was administered i.v. on days 0 and 4 at a dose of 10 mg kg^−1^ day^−1^. Topotecan, cisplatin and etoposide were given at the highest nontoxic dose and cyclophosphamide was given at 90% of the historical LD10 dose, as previously evaluated in athymic mice (Vassal *et al*, 1996; 1997).

### Evaluation of antitumour activity

The activity of each drug was evaluated according to three criteria: (1) the number of complete (CR) and partial (PR) tumour regressions; (2) the TGD; (3) the number of tumour-free survivors (TFS) (Bissery and Chabot, 1991). CR was defined as a tumour regression beyond the palpable limit (15 mm^3^) and PR as a tumour regression exceeding 50% of the initial tumour volume. At least two consecutive tumour measurements had to be observed in order to retain CR and PR. TGD was defined as the difference between the treated group and the control group, within the median time to reach a tumour volume that was five-fold the initial tumour volume (i.e. *time to 5*). For each tumour of the treated groups, the individual TGD was defined as the difference between the individual *time to 5* and the median *time to 5* of the control group. Since the duration of treatment was different from one passage to another, we also considered the TGD, corrected for the duration of treatment (TGDc), which was defined as the difference between TGD and the duration of treatment. TFS were defined as animals that were free of palpable tumour at the end of the experiment (at least 120 days).

### Histological analysis

Xenograft tissue specimens were fixed in acetic acid–formalin–ethanol (Carlo-Erba, Milano, Italy) and embedded in paraffin. The paraffin-embedded sections were stained with haematoxylin–eosin–saffranin for morphology.

### MYCN amplification

*MYCN* copy number was measured using the TaqMan 5′ nuclease fluorigenic real-time quantitative PCR assay, as reported previously (Valent *et al*, 2001).

### Comparative genomic hybridisation (CGH)

CGH was used to evaluate and characterise the genetic anomalies acquired during the establishment of the resistant phenotype. Genomic DNA from sensitive and resistant tumours at passages 1 and 24 was purified using the DNeasy tissue Kit (Qiagen). Hybridisation of differentially labelled tumour and normal DNA to normal metaphase chromosomes was performed using previously published methods (Kallioniemi *et al*, 1992). Digital image analysis was used so that chromosomal regions with abnormal fluorescence ratios could be easily identified. The mean values of individual ratio profiles were calculated from at least 10 metaphases for each tumour specimen. CGH profile shifts were rated as gains and losses if they at least reached the 1.20 and 0.8 thresholds, respectively.

### Protein expression analysis

Crude extracts were prepared from NB xenografts. Frozen tissues (50 mg) were grossly minced, suspended in lysis buffer (containing 0.15 NaCl, 1 mM KH_2_PO_4_, 5 mM MgCl_2_, 1 mM EDTA pH 6.4, 1 mM phenylmethylsulphonyl fluoride, 1 mM dithiothreitol, 1 mM benzamidine, 1 *μ*g ml^−1^ aprotinine, 10 *μ*g ml^−1^ soybean trypsin inhibitor) and homogenised with a potter teflon-glass homogeniser. NaCl (0.55 M) was added and incubated for 1 h on ice for total cell extraction. After centrifugation at 12 000 r.p.m. for 30 min, the supernatant was assayed for topoisomerase I activity. Protein concentration was determined by the BCA method (Pierce). Proteins (50 *μ*g) were separated electrophoretically in 7.5% SDS–polyacrylamide gels and then transferred to a nitrocellulose membrane (Hybond P Amersham Life Science Les Ulis, France). Blots were incubated with human polyclonal topoisomerase I antibody (Topogen INC) diluted at 1 : 8300 followed by the anti-protein A horseradish-peroxidase-conjugated antibody (Amersham Pharmacia Biotech). Detection was performed using a chemiluminescence (ECL) enzyme immunoassay (Amersham Pharmacia Biotech).

### Determination of topoisomerase I catalytic activity

Topoisomerase I catalytic activity of crude extracts was examined by a DNA relaxation assay using supercoiled pHOT1 plasmid DNA as the substrate (Topogen Inc., Columbus, OH, USA). For each sample, 10 extracts were serially diluted in buffer containing 10 mM Tris-HCl, 100 mM Kcl, 1 mM PMSF, and 50 *μ*g ml^−1^ BSA, pH 7.5. Supercoiled DNA (0.5 *μ*g) was incubated with each diluted extract at 37°C for 30 min in 10 × Topoisomerase I assay buffer (Topogen Inc). DNA topoisomers were separated by gel electrophoresis in 1.25% agarose and stained with ethidium bromide. One arbitrary unit of topoisomerase I activity was defined as the amount of topoisomerase I showing relaxation of 0.25 *μ*g DNA under the above-described conditions. Topoisomerase I activity was expressed in arbitrary units (a.u.) per mg of protein.

### ATP-binding cassette (ABC) transporter superfamily analysis

*MDR1* and *MRP1* mRNA expression was analysed by RT–PCR as reported previously (Vassal *et al*, 2003b). The relative expression ratios (RER) were calculated by dividing the fluorescence intensity of the target gene band by that of the GAPDH control gene band. Breast cancer resistance protein (BCRP) analysis was performed by immunohistochemistry. Formalin-fixed paraffin-embedded tumours were cut into 4-*μ*m thick sections and rehydrated. Sections were prepared with the Histo-mouse kit (Zymed) according to the manufacturer's instructions, and incubated for 1 h at room temperature with mouse anti-BCRP BXP-21 monoclonal antibody (Chemicon international) diluted at 1 : 20. Detection was performed using a rabbit anti-mouse IgG horseradish peroxidase conjugate. A further analysis of *BCRP* expression was performed by Northern blotting. Total RNA was prepared using Trizol reagent, according to the manufacturer's instructions. In all, 20 *μ*g of total RNA were fractionated on a 1% agarose–formaldehyde gel and subsequently transferred to a nitrocellulose membrane filter (Hybond N Amersham Life Science Les Ulis, France). Blots were prehybridised for 1 h at 42°C in 5 × SSC (1 × SSC=150 mM sodium chloride, 15 mM sodium citrate, pH 7.0), 5 × Denhardt's solution, 0.2% SDS, 100 *μ*g ml^−1^ salmon sperm DNA and 50% deionised formamide. The blots were then probed using 25 ng of the ^32^P-labelled *BCRP/MXR/ABCP* probe at 42°C overnight. After washing in 1 × SSC/0.1% SDS for 20 min at room temperature and three 10-min washes with 0.2 × SSC/0.1% SDS at 65°C, blots were analysed using a phosphor imaging system (Fujix Bas 2000).

## RESULTS

### Acquisition of *in vivo* resistance to CPT-11

**Figure 1:**
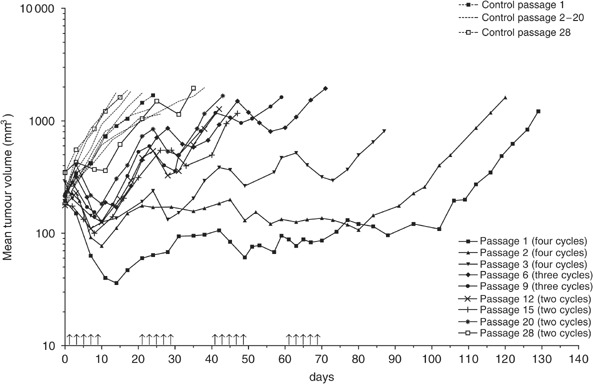
Evolution of the mean tumour volume during 28 consecutive passages. Animals received either saline (dotted line) or CPT-11 at a dose of 27 mg kg^−1^ day^−1^ (full line). Arrows represent the daily i.v injection.

The influence of CPT-11 treatment on the growth of the IGR-NB8 xenografted tumour over 28 passages is shown in Figure 1

. The tumour volume of all untreated controls increased rapidly. During the first three passages, the same total dose of CPT-11 (540 mg kg^−1^) was administered over the same period of time (68 days) and tumour growth displayed the same pattern. Complete regression and PR were observed after the first cycle of treatment. Tumour volumes remained stable during the following three cycles. When treatment was discontinued, tumours remained stable for about 1 month and then started to grow again. Over the first three passages, IGR-NB8 tumour response to CPT-11 was significantly reduced with a TGD from 115 to 69, that is, a TGD corrected for the duration of treatment from 47 days at the first passage to 1 day at the third passage (Table 1

**Table 1:** Antitumour activity of i.v. CPT-11 at dose of 27 mg kg^−1^ day^−1^ against IGR-NB8 xenografts

**Passages**	**Number of cycles**	**Total dose (mg kg^−1^)**	**Treatment duration (days)**	**DT (days)**	**CR^a^**	**PR^a^**	**TGD (days)**	**TGDc (days)**	**TFS**
P1	× 4	540	68	7.1	3/7	4/7	115	47	0
P2	× 4	540	68	8.5	3/14	8/14	97	29	1
P3	× 4	540	68	8.3	2/14	7/14	69	1	1
P6	× 3	405	47	3	0/13	0/13	54	7	0
P9	× 3	405	47	3.7	0/8	0/8	47	0	0
P12	× 2	270	26	4.6	0/7	0/7	29	3	0
P15	× 2	270	26	7	0/10	3/10	27	1	0
P20	× 2	270	26	2.4	0/8	0/8	28	2	0
P28	× 2	270	26	6.1	0/8	0/8	17	0	0

DT=doubling time; TGD=tumour growth delay; TGDc=tumour growth delay corrected for treatment duration; TFS=tumour-free survivors at 120 days.

a CR, PR=complete and partial regression, at first cycle

). In contrast, neither complete or partial tumour regression nor any tumour stabilisation were observed beyond the third passage (Table 1 and Figure 1). At each passage, CPT-11 treatment was stopped as soon as at least 50% of tumours had reached a volume that was five-fold the initial tumour volume. The number of treatment cycles was gradually reduced from 4 to 2, the total dose from 540 to 270 mg kg^−1^ and the duration of treatment from 68 to 26 days from passages 3 to 28. Overall, the TGD was significantly reduced from 115 to 17 days after 28 *in vivo* passages and 68 cycles of treatment. Tumour DT gradually decreased during these 68 cycles of treatment. We had established a NB xenograft that was resistant to CPT-11 (IGR-NB8-R).

### Reverted resistance

**Figure 2:**
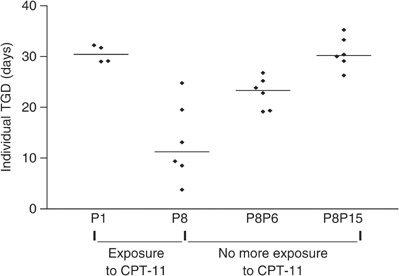
Effect of one cycle of CPT-11 27 mg kg^−1^ day^−1^ × 5. Passage 1 (P1 – sensitive tumour) and passage 8 (P8 – resistant tumour): treatment discontinued in passage 8 (P8). P8P6 and P8P15: resistance verified *in vivo* at P8P6 (P6=6 passages without treatment after passage 8 with treatment); P8P15 (P15=15 passages without treatment after passage 8 with treatment). Central bars: medians.

In order to evaluate the stability of the resistance acquired, IGR-NB8-R tumours at passage 8 were further grown without treatment, while the prolonged treatment process was continued in parallel up to 28 passages. Sensitivity to CPT-11 was checked regularly (every 3–4 passages) by evaluating the TGD after one cycle (27 mg kg^−1^ × 5) of treatment. As shown in Figure 2

, the resistance acquired *in vivo* during the first eight passages was completely reverted after 15 growth passages of IGR-NB8-R without any further exposure to CPT-11. Thus, *in vivo* acquired resistance to CPT-11 was revertible.

### Cross-resistance

**Figure 3:**
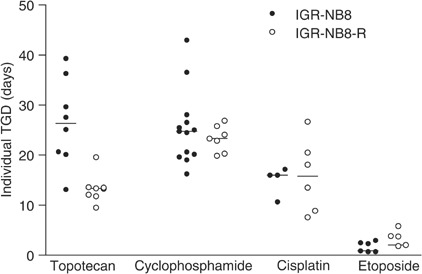
Cross-resistance analysis. IGR-NB8 and IGR-NB8-R xenograft tumours were treated with topotecan (3.2 mg kg^−1^ day^−1^), cyclophosphamide (400 mg kg^−1^), cisplatin (10 mg kg^−1^ day^−1^) and etoposide (20 mg kg^−1^ day^−1^). Central bars: medians.

In order to evaluate cross-resistance to other anticancer drugs *in vivo*, IGR-NB8-R xenografts (between passages 8 and 11) were grown in athymic mice. The sensitivity of the parental tumour (IGR-NB8) and the resistant tumour (IGR-NB8-R) to other anticancer drugs is shown in Figure 3

. IGR-NB8-R and IGR-NB8 displayed similar tumour response to cyclophosphamide, an alkylating agent, (TGD, 24 days) and to cisplatin (TGD, 16 days). Conversely, IGR-NB8-R and IGR-NB8 failed to respond to etoposide, a DNA-topoisomerase II inhibitor. However, IGR-NB8-R was significantly less sensitive to the DNA-topoisomerase I inhibitor topotecan (TGD, 13 days) than the parental IGR-NB8 (TGD, 26 days at 2.7 mg kg^−1^) (Vassal *et al*, 1997). Thus, IGR-NB8-R exhibited cross-resistance to topoisomerase I inhibitors, but not to DNA-damaging agents.

### Characterisation of IGR-NB8-R

**Figure 4:**
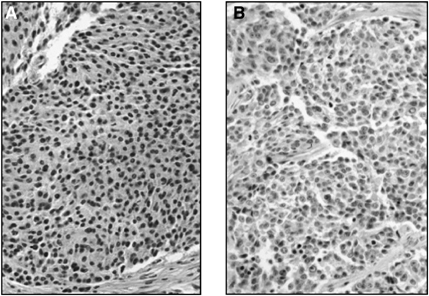
HES staining for morphology of the parental (**A**) at passage 1 and NB resistant to CPT-11, IGR-NB8-R (**B**) xenografts at passage 17. Original magnification × 400.

Fresh tumour tissues were collected from the parental xenograft and from the resistant xenograft at passages 17–20. The parental xenografts displayed the histological features of a poorly differentiated NB composed of undifferentiated neuroblastic cells (small uniform rounded cells) containing a high number of mitotic figures per high-power field and very little schwannian stroma (Figure 4A

). During the first two passages, the stabilisation state was associated with tumour differentiation exhibiting features of a maturing ganglioneuroma (Santos *et al*, 2004) However, the IGR-NB8-R-resistant xenografts showed similar histological features to those observed in the parental xenografts (Figure 4A), namely those of a poorly differentiated NB (Figure 4B). These results indicate that there was no modification of histological features during the acquisition of resistance.

### MYCN amplification

**Figure 5:**
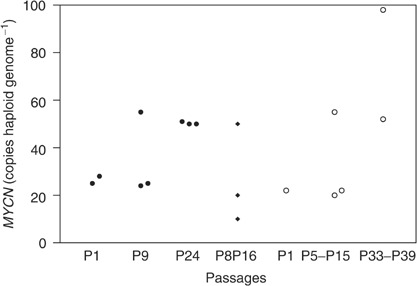
Amplification of *MYCN* oncogene by quantitative real-time PCR. IGR-NB8-R xenograft under exposure to irinotecan [•] at passages 1, 9 and 24; reverted IGR-NB8-R P8P16 [⧫] after 16 passages without treatment with irinotecan; IGR-NB8 xenograft grown in parallel to IGR-NB8-R [○] at P1, between P5–P15 and P33–P39. Each symbol represents one tumour.

Neuroblastomas that overexpress *MYCN* due to amplification of the *MYCN* oncogene are aggressive tumours that become resistant to chemotherapy. High expression of *MRP1* RNA has been reported to be associated with *MYCN* amplification and poor treatment outcomes (Norris *et al*, 1996). Analysis of the *MYCN* oncogene in IGR-NB8-R xenograft showed that in spite of very heterogeneous expression in a given passage, there was no major difference, between *MYCN* amplification at P1 (sensitive tumour), P9 (resistant tumour) and P8P16 (reverted tumour) exhibiting a median value of 26.5, 25, and 20 copies per haploid genome, respectively (Figure 5

). However at passage 24 (resistant tumour), *MYCN* amplification was double that of the other passages studied. A fluorescence *in situ* hybridisation analysis showed that *MYCN* was amplified in double minute form in the nucleus, which explains the heterogeneous amplification observed. This is a current situation observed in primary tumours (Valent *et al*, 2001). Furthermore, in parallel of the 28 passages carried out to obtain the IGR-NB8-R-resistant tumour, the IGR-NB8 xenograft was also maintained during the same period without any treatment. *MYCN* amplification analysis revealed a heterogeneity and a trend to a time-dependent increase in copy number, according to the passage number (Figure 5). Thus, the apparent increase in *MYCN* copies observed in IGR-NB8-R overtime seems to be the consequence of a selective growth advantage procured by increase of the number of copies of *MYCN* rather than directly related to the prolonged exposure to irinotecan.

### CGH analysis

CGH analysis detected the same genomic imbalances in IGR-NB8 at P1 and IGR-NB8-R tumours xenografts at P24: partial chromosome loss was observed at 1p32-pter, 6q25–27, 17p and 22. Partial chromosome gains were observed at chromosome bands 2p23–24, 12p13 and 17q. These genomic imbalances are characteristic in NB cell lines and advanced-stage tumours. However, gains on chromosome 2p24 peaked on IGR-NB8-R, as a result of an increased *MYCN* copy number at passage 24.

### Analysis of topoisomerase I

**Figure 6:**
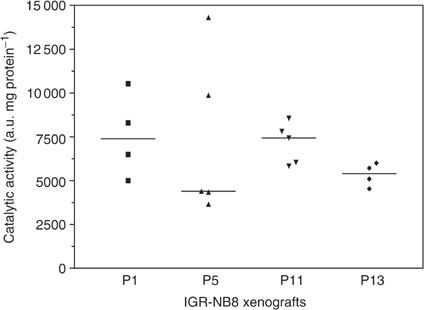
Catalytic activity of topoisomerase I at passages 1, 5, 11 and 13. Each symbol represents one tumour. Horizontal bars: medians.

**Figure 7:**
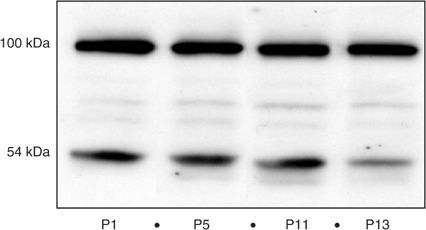
Topoisomerase I analysis by Western blot at passages 1, 5, 11 and 13. Protein from tissue homogenates, 50 *μ*g, were separated in 7.5% SDS–polyacrylamide gels, transferred to a nitrocellulose membrane and immunoblotted with human polyclonal topoisomerase I antibody (Topogen Inc.).

Topoisomerase I catalytic activity was quantified in four to five tumours at passages 1, 5, 11 and 13. (Figure 6

). The average topoisomerase I activity was 7469±2351, 7036±4952, 7410±1405 and 5334±655 a.u. mg^−1^ (mean±s.d.), respectively. The modifications of topoisomerase I catalytic activity was not significant over these passages, while TGD decreased from 115 to 32 days. Moreover Western blot analysis of topoisomerase I showed no difference between the tumours studied (Figure 7

). The human polyclonal anti-topoisomerase I antibody revealed two main bands located at 100 kDa, corresponding to a full-length enzyme and at 54 kDa, a form specific to human tissues (Santos *et al*, 2004) and perhaps the heavy chain of the immunoglobulin molecule (Bronstein *et al*, 1996). Thus, topoisomerase expression and activity was not implicated in the *in vivo* resistance acquisition.

### ABC transporter superfamily analysis

*MDR1* and *MRP1* expression was quantified by RT–PCR before treatment (P1) and at passages 6 (P6) and 11 (P11). Three tumours were studied at each passage. *MDR1*-mRNA expression was detected in all tumour samples. As compared to *GAPDH*-mRNA expression, the *MDR1*-mRNA RER was 1.17±0.46, 1.38±0.43 and 1.23±0.26 at P1, P6 and P11, respectively. These levels of relative expression were comparable with the positive control MCF7^DXR^ 1.44±0.12. *MRP1*-mRNA was also detected in all tumour samples, with an RER of 2.4±0.05, 1.91±0.36 and 2.08±0.75 at P1, P6 and P11, respectively. These levels of relative expression were comparable to that of A549, the positive control (2.86±0.71). These results confirmed the strong basal expression of *MDR1* and *MRP1* in IGR-NB8. However, expression of both *MDR1* and *MRP1* did not increase from P1 to P11, while the TGD was being significantly reduced from 115 to 25 days. Immunohistochemical analysis of BCRP revealed relatively high expression in the positive control (human placenta), and the absence of BCRP expression in sensitive, resistant and chemosensitivity-restored tumours. Northern blotting confirmed these results. *BCRP/MXR/ABCP* mRNA was undetectable in all the tumours analysed at various passages (P1, P3, P7, P12, P18, P21 and P8P15). The positive control (T8 cells) (Maliepaard *et al*, 1999) overexpressing *BCRP* mRNA and *GAPDH* were very positive in all tested samples (data not shown). Thus, CPT-11 resistance *in vivo* in IGR-NB8-R xenograft is not related to MDR1, MRP1 and BCRP expression.

## DISCUSSION

Irinotecan has shown activity against colorectal, oesophageal, gastric, non-small-cell and small-cell lung cancer, leukaemia and lymphomas, as well as central nervous system malignant gliomas (Rothenberg, 2001). One of the major hurdle to clinical development of active agents is intrinsic or acquired chemoresistance. To date, the mechanisms that confer clinical resistance to camptothecin have not been characterised. In the field of oncology, knowledge of drug resistance mechanisms is based largely on *in vitro* studies. Several cell lines selected for resistance to irinotecan have been described and these studies have shed light on mechanisms such as topoisomerase I alteration or increased efflux of irinotecan from the cell (Xu and Villalona-Calero, 2002). However, *in vitro* analyses of resistance mechanisms remain limited. The majority of the variables are controlled and cellular interactions are not taken into account. Moreover, *in vivo* and *in vitro* phenotype of cancer cells do not always coincide. Teicher *et al* derived a series of alkylating agent-resistant variants (*cis*-diamminedichloroplatinum, cyclophosphamide, carboplatin and thiotepa) of the EMT-6 mouse mammary tumour through *in vivo* drug administration to syngeneic BALB/c tumour-bearing mice. The resistance observed was reversible after discontinuation of treatment, as demonstrated in our model. In spite of high levels of resistance *in vivo*, no significant resistance was observed when the cells from these tumours were exposed to the drugs *in vitro*, indicating that a very high level of resistance to anticancer drugs can develop through mechanisms that are exclusively *in vivo* (Teicher *et al*, 1990). Furthermore, Kobayashi *et al* (1993) have suggested that some forms of acquired drug resistance operate only at the multicellular level, as opposed to classic unicellular resistance mechanism.

Thus, our first objective was to establish a subcutaneous NB xenograft model that is resistant to CPT-11. After a series of 68 cycles of CPT-11 treatment and 28 passages *in vivo*, IGR-NB8 showed the characteristics of a CPT-11-resistant tumour. First, no CR or PR was observed after the third passage, whereas the treatment induced 100% of CR and PR at the first passage. Second, during passaging, a very significant decrease (from 115 to 17 days) was observed in the TGD. Third, the number of consecutive cycles that were required for 50% of tumours to attain five-fold their initial volume was reduced from four to two cycles. The CPT-11-resistant IGR-NB8 NB model was designated IGR-NB8-R.

To our knowledge, only one model of *in vivo* acquired resistance to CPT-11 has been reported, which is also an *in vivo* NB (NB-1691) model established by Thompson *et al*. After four rounds of treatment/transplantation (5 mg kg^−1^ administration^−1^), authors observed a resistance to irinotecan and a partial resistance to topotecan. The mechanisms of resistance were not discussed in this paper (Thompson *et al*, 2002, p 541). Our *in vivo* model of acquired resistance evaluated in a therapeutic setting will allow us to confirm and clarify resistance mechanisms observed *in vitro* or to discover new resistance mechanisms by taking into account cellular interactions and thus rendering this approach more realistic from a clinical point of view.

First, IGR-NB8-R was characterised by the absence of crossresistance to DNA-damaging agents and the presence of crossresistance to another topoisomerase I inhibitor, topotecan. Both CPT-11 and topotecan are topoisomerase I inhibitors, which suggests a common resistance mechanism. Altering the topoisomerase I, the common target of these two drugs, can modify treatment efficacy. For example, some camptothecin-resistant cell lines have a mutation in topoisomerase I and this mutation may affect, the catalytic activity of the enzyme (Fujimori *et al*, 1995), or alter interactions with camptothecin or DNA cleavage (Li *et al*, 1996). In our xenograft model, however, qualitative or quantitative modifications of topoisomerase I cannot be responsible for the IGR-NB8-R resistance to CPT-11 and topotecan because no modification of topoisomerase I activity was demonstrated during the analysis with the DNA relaxation assay and the Western blot analysis showed no difference in topoisomerase I expression during the acquisition of resistance. Furthermore, the acquired resistance of IGR-NB8-R was not permanent, since the tumour recovered its initial sensitivity after 15 passages without treatment. This suggests a resistance mechanism that is induced and maintained by CPT-11 and not a phenomenon such as mutation, which is not readily reversible.

We then evaluated the multidrug resistance phenomenon attributed to a change in drug efflux that could explain the cross-resistance of IGR-NB8-R to topotecan and CPT-11. Transmembrane proteins, belonging to the ABC superfamily, participate in energy-dependent drug efflux and confer multidrug resistance. Some transporters in this family, such as MRP and MDR1 that are involved in the active efflux of SN-38 and CPT-11, contribute to resistance to CPT-11 (Chu *et al*, 1999). Furthermore, in NB, it has been reported that high *MRP* RNA expression was associated with *MYCN* amplification and poor treatment outcomes (Norris *et al*, 1996). However, although the basal expression level of *MDR*1 and *MRP*1 was strong in sensitive tumours in our study, it did not increase during the acquisition of resistance. Maliepaard *et al* showed that BCRP, which also belongs to the ATP-binding cassette transporter family, appears to be a highly efficient transporter of topoisomerase I inhibitors in cell lines without overexpression of the multidrug resistance-associated pumps MDR1 and MRP1 (Maliepaard *et al*, 1999). Furthermore, van Hattum *et al* (2002) showed that DX-895, a derivative of camptothecin, is able to induce BCRP protein as a mechanism of resistance in the human ovarian cell line A2780. In our resistant model, which does not overexpress MDR1 or MRP1, we hypothesised that overexpression of BCRP could explain IGR-NB8-R resistance to irinotecan and topotecan without quantitative or qualitative modifications of topoisomerase I activity. This hypothesis prompted us to study BCRP expression; however, like MDR1 and MRP1, no overexpression of BCRP was demonstrated during the acquisition of resistance.

Among the various mechanisms of resistance to irinotecan characterised *in vitro*, we could eliminate those that were usually observed, such as intracellular drug accumulation or drug-target interaction. More recently, nuclear factor kappa B (NF*κ*B) activation was shown to play a role in sensitivity to this drug; treatment with camptothecin was reported to activate NF*κ*B (Wang *et al*, 1999; Cusack *et al*, 2000; Huang *et al*, 2000). Furthermore, the cytotoxic effect of SN-38 was strongly increased through the inactivation of NF*κ*B. The apoptotic response mediated by CPT-11 was dependent on NF*κ*B inhibition, establishing NF*κ*B as a principal mediator of inducible chemoresistance (Wang *et al*, 1999). We are currently exploring the potential role of NF*κ*B in our model of acquired resistance to CPT-11.

We have established a human NB xenograft resistant to CPT-11. This resistance mechanism seems to be novel. It does not imply any of the mechanisms of resistance usually observed in *in vitro* preclinical studies. Furthermore, it is a revertible mechanism that probably does not imply mutations. Our next objective is to investigate the basis of this mechanism of acquired drug resistance. Several genes are likely implicated; therefore, a genomic comparison of the transcriptome of sensitive and resistant tumours with macro- and microarray is in progress. This technology will allow us to rapidly identify differences in expression between genes. Clarifying the resistance mechanism in IGR-NB8-R will allow us to make a step forward in our understanding of acquired chemoresistance and to better target the clinical development of irinotecan.
